# Endoparasites of Wild Mammals Sheltered in Wildlife Hospitals and Rehabilitation Centres in Greece

**DOI:** 10.3389/fvets.2017.00220

**Published:** 2017-12-18

**Authors:** Theophanes K. Liatis, Antonios A. Monastiridis, Panagiotis Birlis, Sophia Prousali, Anastasia Diakou

**Affiliations:** ^1^Laboratory of Parasites and Parasitic Diseases, Faculty of Health Sciences, School of Veterinary Medicine, Aristotle University of Thessaloniki, Thessaloniki, Greece; ^2^Action for Wildlife, Thessaloniki, Greece

**Keywords:** wildlife, mammals, Greece, endoparasites, zoonotic, rehabilitation centres, wildlife hospitals, wildlife–domestic animal interface

## Abstract

Wildlife parasitic diseases represent an important field of investigation as they may have a significant impact on wild animals’ health and fitness, and may also have zoonotic implications. This study aimed to investigate the occurrence of endoparasites in wild mammals admitted to wildlife hospitals and rehabilitation centres in Greece. Sixty-five animals belonging to 17 species and originated from various areas of continental and insular Greece were included in the survey. The most numerous animal species examined were hedgehogs (*n* = 19), red foxes (*n* = 16), and European roe deer (*n* = 6). Faecal samples were collected individually and examined by floatation and sedimentation method. Parasites were found in 46 (70.7%) of the animals. Most parasites found in canids, felids, and ruminants are of great relevance to the domestic animals’ health and some of them are also of zoonotic importance. To the best of the author’s knowledge, this is the first report of endoparasites in hedgehogs, roe deers, fallow deers, badgers, and bats, and the first report of the pulmonary nematode *Troglostrongylus brevior* in a wild cat in Greece. The significance of the parasites found in each animal species in regard to their health and their relevance to domestic animals and human health is discussed.

## Introduction

The importance of wildlife protection and conservation came under the spotlight several decades ago and today is more comprehensive and integrated than ever (1). Wildlife infectious diseases are a key field of investigation with regard to robustness and conservation of wild animal populations, but are also important as some of them may also affect domestic animals and where zoonotic pathogens are involved, humans too. The study of such interactions constitutes the core of the One Health approach.

Parasitic diseases are among the most prevalent and important infectious diseases in wildlife. As far as free ranging and wild animals are concerned, parasitism is the norm rather than the exception. Although almost always present, parasites are usually in balance with the host organism, doing little damage or having little clinical impact (2, 3). As accurately described before, in every evaluation of parasitism in wild animals three main points should be considered: the effect and importance of the parasites on the hosts themselves, the transmissibility of parasites to domestic animals, and the relationship to public health (2).

For areas with large populations of free roaming dogs and cats and open type herds of productive animals, wildlife and domestic animal interactions and spillovers of parasitic infections are very likely (4). Both of the abovementioned conditions are being met in Greece. Greek wildlife is estimated to include 102 terrestrial mammal species (5). Urbanisation, poisoned baits, land use change, hybridism, and fires are among the most significant threats for wild animals in Greece, where many species are characterised as endangered (5). At present, there are relatively few wildlife veterinary hospitals and rehabilitation centres, which are not fully organised or funded by the state, but function mostly as non-governmental organisations or citizens’ associations, supported exclusively by volunteers. Most of wild animals are admitted to these hospitals due to injuries, disorientation, orphanage, car accidents or infectious and parasitic diseases. The major role of such centres is to preserve the national biodiversity through individual wild animal treatment and rehabilitation as well as to provide animal welfare during their convalescence. At the same time, these organisations support research to study different aspects of wildlife biology, nutrition, disease epizootiology and other biomedical factors of scientific interest.

Parasites and parasitic diseases of wild mammals in Greece have been studied to a relatively small extent, in individual cases or confined animal populations (6–12). Globally, one of the most significant challenges of carrying out a wildlife parasitological survey is the collecting of biological material from wild animals, which is usually not an easy task. Wildlife hospitals provide access to such samples from various animal species, which in most cases are species with closer interaction with domestic animal and human populations and are thus of great interest as far as the transmission of parasites between these groups of hosts is concerned. In this context, this study aimed to investigate the occurrence of endoparasites in wild mammals admitted to wildlife hospitals and rehabilitation centres in Greece and thus to evaluate the possibility of transmission of parasites among wildlife and domestic animals and/or humans.

## Materials and Methods

### Bioethics

This study was carried out in accordance with the recommendations of the Presidential Decree 56/13 “Bringing Greek legislation into line with Directive 2010/63/EC of the European Parliament and of the Council of 22nd September 2010 (L 276/33/20.10.2010) regarding the protection of animals used for experimental and other scientific purposes” that does not require approval by ethics committee, as it did not include any experimental practices on the animals, or practices likely to cause pain, suffering, distress or lasting harm equivalent to, or higher than, that caused by the introduction of a needle in accordance with good veterinary practice.

### Study Period and Animal Species

From September 2012 to October 2017, 65 mammals submitted to two wildlife hospitals and rehabilitation centres, “Action for Wildlife” based in Thessaloniki and “EKPAZ – Hellenic Centre for Wildlife Rescue and Rehabilitation” based on the island of Aegina were included in the survey. The animals originated from various areas of continental and insular Greece and were admitted to the hospitals as victims of road accidents, due to injuries, mobility restriction, shock, poisoning, confusion, weakness and exhaustion, disorientation or orphanage and illegal capture. The mammals examined belonged to the following 17 species (in order of number of animals examined): European hedgehog (*Erinaceus europaeus, n* = 19), red fox (*Vulpes vulpes*, 16), European roe deer (*Capreolus capreolus*, 6), red squirrel (*Sciurus vulgaris*, 4), brown hare (*Lepus europaeus*, 3), grey wolf (*Canis lupus*, 3), beech marten (*Martes foina*, 2), Eurasian badger (*Meles meles*, 2), Eurasian otter (*Lutra lutra*, 2), golden jackal (*Canis aureus*, 1), fallow deer (*Dama dama*, 1), European wild cat (*Felis silvestris silvestris*, 1), European mink (*Mustela lutreola*, 1), coypu (*Myocastor coypus*, 1), common noctule bat (*Nyctalus noctula*, 1), greater noctule bat (*Nyctalus lasiopterus*, 1), and brown rat (*Rattus norvegicus*, 1) (Table 1). For each animal, a record including available data such as area where the animal was found, reason for admission, clinical signs, sex, and age, was kept.

**Table 1:** The examined species and number of mammals from wildlife.

Animal order	Total *n*	*n* of Positive	Parasites (*n* of positive animals)
Animal species			
**Carnivora**			
*Vulpes vulpes*	16	16	Ancylostomatidae (8) *Brachylaima* spp. (5) *Eucoleus aerophilus* (5) *Trichuris vulpis* (5) Acanthocephala (4) *Toxocara canis* (3) *Diplopylidium/Joyeuxiella* spp. (2) *Alaria alata* (1) *Cystoisospora canis* (1) *Sarcocystis* spp. (1) Trematoda (1)
*Canis lupus*	3	3	*Cystoisospora rivolta* (2)
*Canis aureus*	1	1	Ancylostomatidae (1) *E. aerophilus* (1) *Sarcocystis* spp. (1) *T. canis* (1)
*Felis silvestris silvestris*	1	1	*Cystoisospora felis* (1) *Toxocara cati* (1) *Troglostrongylus brevior* (1)
*Lutra lutra*	2	0	–
*Martes foina*	2	0	–
*Meles meles*	2	1	*Eimeria* spp. (1)
*Mustela lutreola*	1	0	–

**Lagomorpha**			
*Lepus europaeus*	3	2	*Trichostrongylys* spp. (2) *Dicrocoelium dendriticum* (1) *Eimeria* spp. (1) *Linguatula serrata* (1)

**Artiodactyla**			
*Capreolus capreolus*	6	4	*D. dendriticum* (2)
*Dama dama*	1	1	*Capillaria bovis* (1) *Eimeria* spp. (1)

**Rodentia**			
*Myocastor coypus*	1	0	–
*Rattus norvegicus*	1	0	–
*Sciurus vulgaris*	4	0	–

**Eulipotyphla**			
*Erinaceus europaeus*	19	15	*Crenosoma striatum* (9) *Capillaria* spp. (7) *Physaloptera clausa* (6) Acanthocephala (3) *Eimeria* spp. (3) *Brachylaima* spp. (1) *Hymenolepis erinacei* (1) Trematoda (1)

**Chiroptera**			
*Nyctalus noctua*	1	1	Capillariinae (1) Trematoda (1)
*Nyctalus lasiopterus*	1	1	Capillariinae (1) Trematoda (1) Trichostrongyloidea (1)

Total	65	46	

### Parasitological Methods

Faecal samples were collected from the boxes or cages where the animals were kept individually and were preserved at 4–7°C until their transfer to the laboratory. In the case of a hare, the whole gastrointestinal tract and liver were examined after the death of the animal.

The faecal samples were examined by two standard parasitological methods, i.e., zinc sulphate (ZnSO_4_) flotation and merthiolate iodine formaldehyde (MIF)—ether sedimentation (13, 14). In brief, for the flotation method, approximately 1 g of faecal material was diluted with tap water and passed through a sieve (No. 150) into a centrifuge tube. The tube was centrifuged at 200 × *g* for 3 min, the supernatant fluid was discharged down to approximately 1 cm above the sediment and zinc sulphate (ZnSO_4_·7H_2_O) solution 33.2% (w/v) was added to the sediment. After thorough dilution of the sediment, zinc sulphate solution was added to just over the top of the tube and a cover slip was placed on the top of the tube. After centrifugation at 150 × *g* for 1 min, the cover slip was carefully removed and placed on a microscope slide. For the sedimentation method, approximately 1 g of the faecal material was diluted in MIF solution, passed through a sieve (No. 150) into a centrifuge tube, 5 ml ether was added, and the content of the tube was homogenised by vigorous shaking. After centrifugation at 200 × *g* for 3 min, all the phases of the centrifuged material, except the sediment, were discharged. Drops of the sediment were placed on a microscope slide and covered with a cover slip. All the preparations were examined under the optical microscope at 100×, 400×, and 1,000× magnification.

The identification of the parasitic stages (eggs, larvae, cysts, and oocysts) found was based on morphological criteria (15–17).

### Follow-up of the Cases

Antiparasitic treatment was administered to all of the animals that were found positive for parasites. However, in most cases, follow-up was not feasible, thus evaluation of the antiparasitic treatment was not performed.

### Statistical Analysis

Results were statistically analysed with the chi-square test of independence and the Fisher’s exact test (*p* < 0.05) with the software IBM SPSS Statistics 23. The parameters analysed were the age of the animal (young vs adult), the area where the animal was found (Northern vs Southern Greece), the clinical condition (weakness/exhaustion vs other clinical condition), and sex, in association with positive result in the parasitological examinations.

## Results

Endoparasites were found in 46 (70.7%) out of 65 animals examined, and the results are summarised in Table 1. The animal species examined belong to six orders, i.e., Carnivora, Lagomorpha, Artiodactyla, Rodentia, Eulipotyphla, and Chiroptera. The parasites found were identified to the genus or species level (21 genera/species) and in some cases to the phylum, superfamily, or subfamily level (Acanthocephala, Trematoda, Capillariinae, and Trichostrongyloidea in 7, 4, 2, and 1 cases respectively).

More precisely, in the order of Carnivora, all (*n* = 16) red foxes (*V. vulpes*) examined were found to be infected with one or more parasites. The parasites found were Ancylostomatidae (in eight animals), *Eucoleus aerophilus* (*n* = 5), *Trichuris vulpis* (*n* = 5), *Brachylaima* (*Brachylaemus*) spp. (*n* = 5), Acanthocephala (*n* = 4), *Toxocara canis* (*n* = 3), *Diplopylidium/Joyeuxiella* spp. (*n* = 2), *Cystoisospora canis* (*n* = 1), *Sarcocystis* spp. (*n* = 1), *Alaria alata* (*n* = 1), and Trematoda (*n* = 1). Similarly, all three grey wolves (*C. lupus*) examined were infected; *Cystoisospora rivolta* was found in two animals and *Sarcocystis* spp. in 1. In the only golden jackal (*C. aureus*) examined, a mixed infection with *E. aerophilus, T. canis*, and Ancylostomatidae was found. Finally, in the wild cat *F. silvestris silvestris, Toxocara cati, Troglostrongylus brevior*, and *Cystoisospora felis* was found. Of the rest of the carnivores examined, only one Eurasian badger (*M. meles*) was found infected with *Eimeria* spp.

In the three brown hares (*L. europaeus*) examined, the parasites *Trichostrongylys* spp. (*n* = 2) and *Eimeria* spp. were found in the faecal examinations. Furthermore, *Dicrocoelium dendriticum* adults and *Linguatula serrata* larvae were found in the post mortem examination of the gastrointestinal tract and liver of one hare.

In the representatives of the Artiodactyla, *D. dendriticum* was found in two of the four European roe deer (*C. capreolus*) examined, and *Capillaria bovis* and *Eimeria* spp. were found in fallow deer (*D. dama*).

The most numerous animal species examined was the hedgehog (*E. europaeus*, 19 animals), where *Crenosoma striatum* (in 9 animals), *Capillaria* spp. (*n* = 7), *Physaloptera clausa* (*n* = 6), *Eimeria* spp. (*n* = 3), Acanthocephala (*n* = 3), *Brachylaima* spp. (*n* = 1), *Hymenolepis erinacei* (*n* = 1), and Trematoda (*n* = 1) were found.

In the two species of bats (Chiroptera) examined eggs of Capillariinae (in two animals), Trematoda (*n* = 2), and Trichostrongyloidea (*n* = 1) were found. Finally, parasites were not found in any of the rodents (*M. coypus, R. norvegicus*, and *S. vulgaris*) examined.

**Figure 1:**
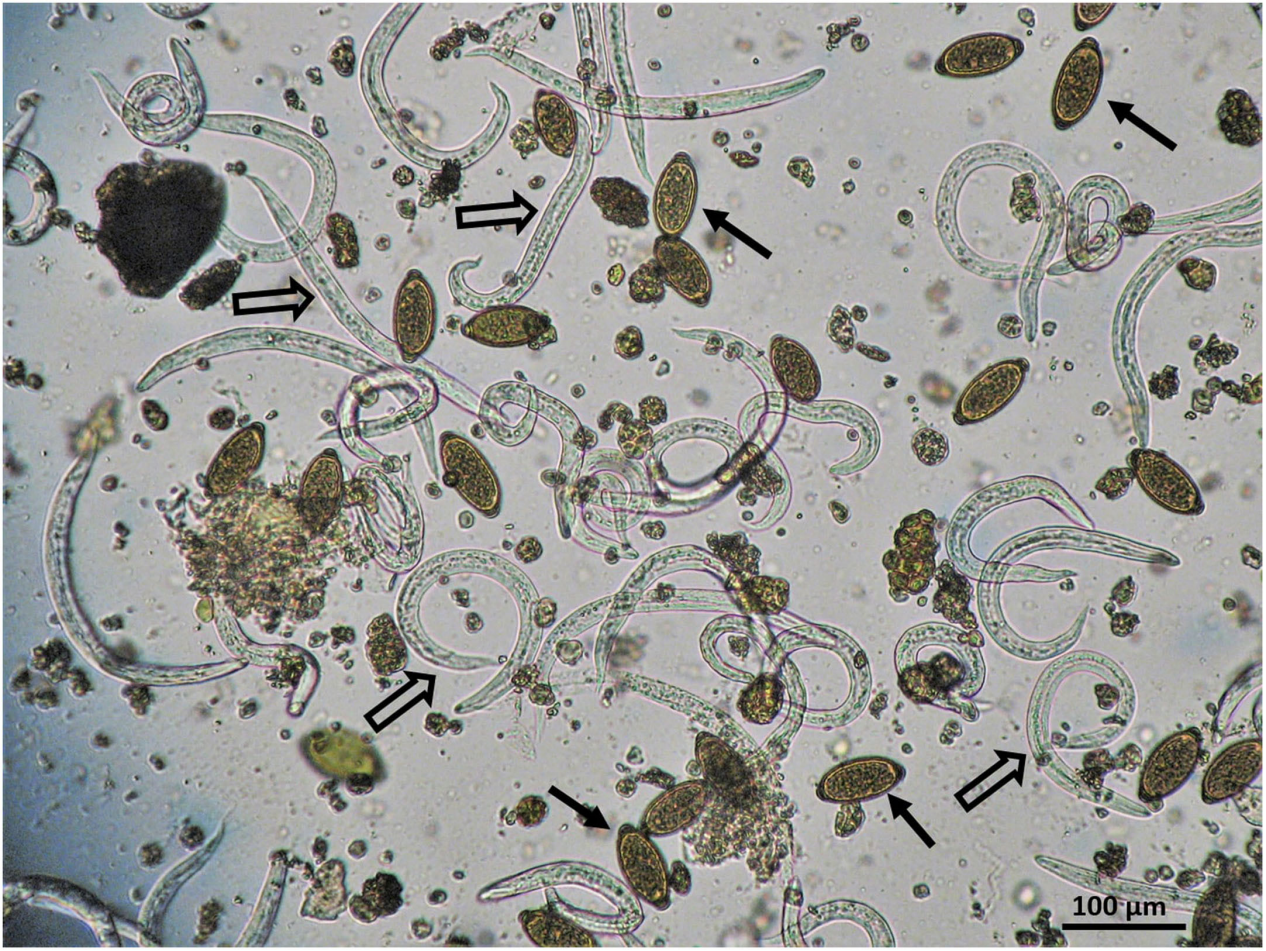
Mixed infection by *Crenosoma striatum* (empty arrows) and *Capillaria* spp. (black arrows) in a hedgehog hospitalised in a wildlife hospital in Greece.

Mixed infections were common (Figure 1) and were recorded in 27 animals. More precisely, five different parasites were found in a fox, four in two hedgehogs, three in five foxes, two hedgehogs and a jackal, two in six hedgehogs, five foxes, and in a hare, the wild cat, the fallow dear, and both bats.

According to the results of the statistical analysis, there was no statistically significant association between the factors evaluated with the presence of parasites. More precisely, the calculated *p* values were 0.729, 1.000, 0.655, and 0.693 for age, area, clinical condition, and sex, respectively.

## Discussion

Knowledge of parasite fauna in wildlife is limited (18, 19). However, the increase in studies and efforts around the One Health concept recently underlines the importance of investigation and understand the impact of parasites in wild animals, domestic animals, and humans. This objective requires constant surveillance and extension of investigation to new areas, biotopes, and animals’ species (3). To the best of the authors’ knowledge, this is the first report of endoparasites in hedgehogs, roe deer, fallow deer, badgers, and bats in Greece.

It is obvious that the parasites of certain wild animals, such as canids, felids, and ruminants, are generally of greater relevance to the domestic animals’ health, because of their taxonomic relation and consequently the existence of common parasitic species. On the other hand, most parasites in animals such as hedgehogs or bats are significant in the context of conservation, robustness, and welfare of these wild populations. The majority of the parasites found in the group of carnivores can be transmitted to the domesticated carnivores, i.e., dogs and cats and *vice versa*. Most importantly, some of these parasites are additionally of zoonotic importance.

*Toxocara* spp. found in carnivores here is the most common intestinal parasite of dogs and cats (20). *Toxocara* spp. infection in animals can be accompanied by severe clinical signs, such as coughing, diarrhoea, vomiting, and even intestinal rapture (21). Most importantly, *Toxocara* spp. may infect humans and is the agent of human toxocarosis, one of the most common parasitic zoonoses worldwide, that is associated with larva migrans syndrome (22).

Ancylostomatidae were found here in foxes and the jackal. These parasites include the common hookworms of carnivores, i.e., *Uncinaria stenocephala* and *Ancylostoma caninum* (15). These nematodes and especially *A. caninum* are quite pathogenic and can cause developmental impairment and some severe clinical signs such as diarrhoea, anaemia, and hypoproteinaemia which can eventually lead to death (23). The prevalence of infection by these parasites in dogs has recently been found to be 7.4% in Poland (20) and 33% in Romania (24). Furthermore, these nematodes can cause enteritis, skin lesions and cutaneous larva migrans syndrome in humans (25–27) and impact human health.

*Trichuris vulpis*, found in five of the foxes examined here, is a nematode parasite of the large intestine of canids. It is a common finding in foxes: for example, 33 and 17% of the foxes in Tunisia and Netherlands, respectively, have been found infected with this nematode (28, 29). *Trichuris vulpis* is also often found in dogs. For example, in some recent surveys it has been found in 8.4 and 10% of dogs in Poland and Slovak Republic, respectively (20, 30) and in 6.4% in Kennel dogs of Northern Belgium (31). This parasite produces particularly environment resistant eggs, has a direct life cycle and can cause severe colitis, with lesions like thickening, ulceration, and necrosis of the intestinal mucosa, severe anaemia, dehydration, and even death of the animal (32). Although there have been some reports in the past connecting *T. vulpis* with patent infection and visceral larva syndrome in humans, the zoonotic potential of this parasite is uncertain and needs further clarification, thus is currently not considered zoonotic (32).

*Alaria alata* was found in one of the foxes examined. This is an important finding as this trematode is the agent of alariosis, a new emerging food-borne disease of humans (33). Little information exists about alariosis that is caused by the migration of *A. alata* larval stage (mesocercariae). This trematode has various carnivores as definitive hosts, and two intermediate hosts: an aquatic gastropode and an amphibian. However, the large number of paratenic hosts that have been identified are important in this parasite’s epizootiology/epidemiology. Regarding human infection, the most important paratenic host is considered to be the wild boar, as human infection occurs when raw or undercooked infected meat is consumed (33). Foxes are an important reservoir of the parasite as they can be infected in high percentages. For example, recently, 26 and 49% of foxes were found to be infected in Ireland and Hungary, respectively (34, 35).

*Troglostrongylus brevior* is a pulmonary nematode that is considered primarily a parasite of wild cats (*F. silvestris silvestris*) (36). It is noteworthy that 71.4% of wildcats examined in southern Italy were found infected by this parasite (37). However, in recent years, *T. brevior* has become a parasite of emerging significance in the domestic cat (*F. s. catus*) also (38). Although *T. brevior* has already been found in some areas where currently wild cats are non-existent (39, 40), in most cases, the distribution pattern of this parasite is compatible with the distribution of the wild cat (41, 42). Moreover, the genetic characterisation of various isolates of *T. brevior*, both from wild cats and domestic cats (43) confirmed that these animal species share common lungworm populations (same haplotypes), thus may be involved in a common transmission cycle. Consequently, the finding of *T. brevior* in the wild cat examined in this study constitutes the first report of this parasite in wild cats in Greece, and it is an indication that the wild cat–domestic cat circulation of the parasite is also a realistic scenario in the examined area.

*Eucoleus aerophilus* (syn. *Capillaria aerophila*) is a nematode of the respiratory tract that can affect both canids and felids and was found in 5 of the 16 foxes and in the single jackal examined in this study. *Eucoleus aerophilus* is considered one of the most common lungworms in wild carnivores in Europe. For example, it was found in 33.3% of wild cats in southern Italy (37), in 30% of red foxes in Pyrenees area (44), and in 84% of red foxes in Serbia (45). Its presence in wildlife is important because it can also affect cats and dogs (46) and moreover, because it is potentially zoonotic with occasionally severe implications for human health (agent of human capillariosis) (47).

The cestodes *Diplopylidium/Joyeuxiella* spp. found in two foxes are common parasites of wild and domestic carnivores. The transmission from one animal to another occurs *via* various intermediate and paratenic hosts (arthropods and small vertebrates). Although generally infection by cestodes remain subclinical, it is occasionally accompanied by signs related to intestinal function disorders (48).

The coccidian parasite *Sarcocystis* spp. in carnivores (definite host) develops in the epithelial cells of the intestine, usually causing little damage (asymptomatic infection) and is spread to the environment *via* faeces. In herbivores, omnivores or birds (intermediate host) the parasite develops in the muscles, where in some cases it can cause important lesions (15). In countries such as Greece, where open type flock rearing (mostly sheep and goats) is practiced, the infection of wild and free ranging carnivores with *Sarcocystis* spp. represents a significant threat for animal production, as in some cases it leads to abattoir condemnation of meat during veterinary inspection due to excessive infection with the parasite.

Apart from *Sarcocystis*, other Coccidian protozoa found were *Cystoisospora* spp. and *Eimeria* spp. *Cystoisospora canis, C. felis*, and *C. rivolta* infect canids, felids, and both animal families, respectively, while *Eimeria* spp. are parasites of a large variety of animal species. These parasites have a direct life cycle, are transmitted *via* contaminated soil and/or water, and may cause severe enteritis characterised by diarrhoea, anorexia, weight loss, vomiting, and even death, especially in young individuals (49). Wildlife and domestic animals in many cases share common parasites of these genera, thus the transmission cycles can be practically considered merged.

Nymphs of the pentastomid parasite *Linguatula serrata* were found on the intestines of an examined hare. This is a rather common finding in this animal species as it was found on the intestinal serosa in 19% of the hares examined in Northern Greece (12). Carnivores are the definitive host of this parasite and in wolves in Central Greece, eggs of the parasite were found in 18.4% of the samples examined (10). Meat producing animals, such as sheep and goats are also intermediate hosts of *L. serrata*, and the percentage of infection in some areas (50) may have an important impact on the economy of animal production, as evidence exists that *L. serrata* nymphs transfer bacteria that contaminate offal, enhancing post mortem spoilage of the infected organs (51). In addition, this parasite can have a significant impact on human health, causing the so-called marrara or halzoun syndrome, characterised by inflammation and itching of the upper respiratory tract (52, 53).

Some of the parasites found in herbivorous animals in this study are also very common in domestic animals. *Dicrocoelium dendriticum*, a trematode parasite of the biliary ducts, was found in roe deer and hare. *Capillaria bovis*, a nematode parasite of the small intestine in many species of wild and domestic ruminants (54–56) was found in fallow dear in this study. The nematode of the genus *Trichostrongylus* found in hares here was very likely *T. retortaeformis* as it was the only species of this genus found in necropsied hares in Greece (12). This nematode can also infect domestic rabbits. All the abovementioned animals may represent reservoir hosts of these parasites and their epizootiological role in the infection of domestic herbivores—especially sheep and goats—because of the open type flock rearing may be decisive.

Acanthocephala were found in foxes and hedgehogs. These parasites generally cannot be identified to a lower than the phylum level by coprological examinations, as their eggs are similar in morphology in many genera and species. Moreover, in carnivores, pseudoparasitism is a common finding, as their prey may be infected by such parasites. For example, in two cases in this study, morphologically similar acanthocephalan eggs were found both in foxes and their common prey, hedgehogs. A second faecal sample from these foxes a number of days later would most likely have solved this problem, but could not be collected as these foxes were released very soon after their admission to the shelter. Some species of Acanthocephala reported in foxes are *Macracanthorhynchus hirudinaceus, Macracanthorhynchus catulinus, Prosthorhynchus transversus, Oncicola canis*, and *Pachysentis* spp. (11, 28, 57) and in hedgehogs *Nephridiacanthus major* and *Plagiorhynchus* (*Prosthorhynchus*) *cylindraceus* (58, 59). Regarding Acantocephala and their relation to predator–prey relationships, the phenomenon of postcyclic transmission, where adult parasites when ingested within their definite host–prey survive and parasitise the predator, should also be taken into consideration as a way of encountering the same parasite species both in prey and predators (60).

Pseudoparasitism, as mentioned earlier, could also be involved in the occurrence of *Brachylaima* spp. in both foxes and hedgehogs. *Brachylaima erinacei* is quite common in European hedgehogs (61) and although foxes may be parasitised by other species of this genus like *Brachylaima recurva* and *Brachylaima tokudai* (57, 62), the presence of *Brachylaima* eggs in the faeces of these animals could also be attributed to pseudoparasitism after preying on infected hedgehogs.

The lung worm *C. striatum*, transmitted by snails and slugs (intermediate hosts) was the most commonly found parasite in hedgehogs (in 9 of the 19 animals). Furthermore, the stomach nematode nematode *P. clausa* was found in six animals. This parasite has an indirect life cycle, with insects as intermediate and reptiles as paratenic hosts. *Crenosoma striatum* and *P. clausa* are particularly common in hedgehogs and can parasitise practically all individuals in some areas (63). Mixed infections by these parasites were observed in 43% of the hedgehogs examined in Iran (63). Similarly, this particular mixed infection was recorded in five of the animals in the present survey. In addition, *Capillaria* spp., *Eimeria* spp. *H. erinacei*, and non-identified trematodes were found in hedgehogs in the present survey. Hedgehogsare known to harbour a large variety of parasites, probably due to their omnivorous diet that includes, apart from of fruits and grass, animals that serve as intermediate or paratenic hosts of various parasites, i.e., earthworms, gastropods, beetles, reptiles, amphibians, fish, rodents, and birds (63, 64). In the case of heavy parasitism, these animals may show severe clinical signs or even die and also may act as a transport and reservoir host for parasites in urban, semi-urban, and rural environments (59), especially as these animals live mostly in close contact with humans and domestic animals (65).

Eggs of Capillariinae, Trichostrongyloidea, and Trematoda were found in the two representatives of Chiroptera examined in this study. Very few surveys of endoparasites of bats exist worldwide. For example, some endoparasites of the abovementioned taxonomic groups found in bats of the genus *Nyctalus* in Japan are the capillarian *Capillaria pipistrelli*, the trematodes *Plagiorchis muris* and *Mesodendrium macrostomum*, and the trichostrongyloid *Molinostrongylus skrjabini longispicula* (66). Although some pathogens of bats and viruses particularly are well studied with regard to their impact on the same animal, on other animal species and on humans (67), analogous surveys on endoparasites are insufficient or non-existing and thus need to be planned and materialised.

It is worth noting that no endoparasites were found in the order of Rodentia. As the group was constituted basically by squirrels, this result was rather expected, as it has been repetitively found that Sciuridae have a relatively poor parasitic fauna (6).

Interestingly, none of the parameters examined (age, sex, area, and clinical condition) were statistically associated with parasitic infection. However, a highest percentage of infection (88.9%) was found in the group of animals showing exhaustion and weakness, while the animals with injury (car accident or dog attack) and the orphans were infected to a lower percentage, i.e., 75 and 50%, respectively. This observation could be attributed to opportunistic establishment of parasites in the weakened organism of these animals and/or to the pathogenetic mechanisms of the parasites that had an impact on the health status of these animals, resulting in their weakness. Either way, parasitism would most likely complicate the health problems of these animals and threaten their recovery in the absence of antiparasitic treatment (2).

It is indeed true that our knowledge of the kind of infectious pathogens that occur in wild animals and which of these could actually be transmitted and established in domestic animals or humans is very limited (3). For that reason, epizootiological investigations are particularly important and should be conducted systematically, both cross-sectionally and longitudinally, and also in an attempt to cover as many areas, ecosystems and biotopes as possible. Such surveillance can provide the scientific community with important insights regarding host–parasite relationships, prevalence, and transmission patterns.

Furthermore, it is also important to mention that although faecal examinations—the most practical way of investigating endoparasites in live animals—are very useful, as they simultaneously inspect both the digestive and the respiratory systems for metazoa and protozoa, necropsy usually shows higher sensitivity in revealing the metazoan parasitic fauna and should be implemented whenever possible (68).

Finally, molecular analyses of parasites obtained from wildlife are of particular importance and should be implemented in future surveys, whenever feasible, as these can provide identification of the parasites to a level that would reveal paths of transmission among animal populations (wild and domestic) and clarification of their zoonotic potential (3).

## Author Contributions

TL conceived and designed the study, reviewed the data, performed parasitological examinations, and wrote the first draft of the manuscript. AM and PB performed parasitological examinations. SP performed the clinical procedures (history, physical examination, nursing, and treatment) and provided the samples. AD designed the study, reviewed and interpreted the data, performed parasitological examinations, and carried out the major editing of the manuscript. All the authors critically reviewed the manuscript.

## Conflict of Interest Statement

The authors declare that the research was conducted in the absence of any commercial or financial relationships that could be construed as a potential conflict of interest.
